# External Quality Assessment (EQA) for SARS-CoV-2 RNA Point-of-Care Testing in Primary Healthcare Services: Analytical Performance over Seven EQA Cycles

**DOI:** 10.3390/diagnostics14111106

**Published:** 2024-05-26

**Authors:** Susan J. Matthews, Kelcie Miller, Kelly Andrewartha, Melisa Milic, Deane Byers, Peter Santosa, Alexa Kaufer, Kirsty Smith, Louise M. Causer, Belinda Hengel, Ineka Gow, Tanya Applegate, William D. Rawlinson, Rebecca Guy, Mark Shephard

**Affiliations:** 1 International Centre for Point-of-Care Testing, Flinders University, Bedford Park, SA 5042, Australia; kelcie.miller@flinders.edu.au (K.M.); kelly.andrewartha@flinders.edu.au (K.A.); melisa.milic@flinders.edu.au (M.M.); mark.shephard@flinders.edu.au (M.S.); 2 Royal College of Pathologists of Australasia Quality Assurance Programs, St. Leonards, NSW 2065, Australia; deane.byers@rcpaqap.com.au (D.B.); peter.santosa@rcpaqap.com.au (P.S.); alexa.kaufer@rcpaqap.com.au (A.K.); 3 Kirby Institute, UNSW, Sydney, NSW 2052, Australia; ksmith@kirby.unsw.edu.au (K.S.); lcauser@kirby.unsw.edu.au (L.M.C.); bhengel@kirby.unsw.edu.au (B.H.); igow@kirby.unsw.edu.au (I.G.); rguy@kirby.unsw.edu.au (R.G.); 4 Serology and Virology Division (SAViD), SEALS Microbiology, NSW Health Pathology, Prince of Wales Hospital, Sydney, NSW 2031, Australia; w.rawlinson@unsw.edu.au; 5 School of Medical Science, University of NSW, Sydney, NSW 2052, Australia; 6 School of Clinical Medicine, University of NSW, Sydney, NSW 2052, Australia; 7 School of Biotechnology and Biomolecular Sciences, University of NSW, Sydney, NSW 2052, Australia

**Keywords:** quality assessment, quality assurance, severe acute respiratory syndrome coronavirus 2 (SARS-CoV-2), COVID-19, ribonucleic acid (RNA), analytical performance, Point-of-Care (POC) testing, primary healthcare, remote, first nations communities

## Abstract

In April 2020, the Aboriginal and Torres Strait Islander COVID-19 Point-of-Care (POC) Testing Program was initiated to improve access to rapid molecular-based SARS-CoV-2 detection in First Nations communities. At capacity, the program reached 105 health services across Australia. An external review estimated the program contributed to averting between 23,000 and 122,000 COVID-19 infections within 40 days of the first infection in a remote community, equating to cost savings of between AU$337 million and AU$1.8 billion. Essential to the quality management of this program, a customised External Quality Assessment (EQA) program was developed with the Royal College of Pathologists of Australasia Quality Assurance Programs (RCPAQAP). From July 2020 to May 2022, SARS-CoV-2 EQA participation ranged from 93 to 100%. Overall concordance of valid EQA results was high (98%), with improved performance following the first survey. These results are consistent with those reported by 12 Australian and 4 New Zealand laboratories for three SARS-CoV-2 RNA EQA surveys in March 2020, demonstrating that SARS-CoV-2 RNA POC testing in primary care settings can be performed to an equivalent laboratory analytical standard. More broadly, this study highlights the value of quality management practices in real-world testing environments and the benefits of ongoing EQA program participation.

## 1. Introduction

Early in the COVID-19 pandemic, the National Aboriginal Community Controlled Health Organisation (NACCHO) and the Australian Government (Department of Health and Aged Care) recognised the serious health risk that the novel coronavirus (SARS-CoV-2) posed to First Nations communities living across Australia’s diverse, 7,688,287-kilometre (km)^2^ geographical footprint. Of major concern were remote First Nations communities, where access to rapid and accurate COVID-19 testing was poor and long distances to laboratory facilities likely delayed diagnostic results for periods of up to several days, further exacerbating community transmission risk. At this time, prompt detection and quarantining of COVID-19-positive cases were essential disease control strategies as the scientific understanding of severe acute respiratory syndrome coronavirus 2 (SARS-CoV-2) was emerging, and rapid diagnostic tests, preventative vaccinations and anti-viral treatments for COVID-19 were only starting to be conceptualised and/or developed globally.

On this basis, in April 2020, the Australian Government funded the Kirby Institute (University of New South Wales) in partnership with the Flinders University International Centre for Point-of-Care Testing (FUICPOCT) to implement the Aboriginal and Torres Strait Islander COVID-19 Point-of-Care Testing Program (hereafter referred to as the ‘COVID-19 POC Testing Program’) to improve access to rapid and accurate COVID-19 detection in remote First Nations communities across Australia [1]. Leveraging existing community collaborative partnerships, device and information technology infrastructure, local operator training and competency resources and analytical quality management processes developed from a previous randomised controlled trial [2] and translational research program [3] for sexually transmitted infection molecular-based point-of-care (POC) testing in remote primary care, SARS-CoV-2 ribonucleic acid (RNA) POC testing in the program promptly commenced in May 2020. The COVID-19 POC Testing Program expanded quickly and the onsite SARS-CoV-2 RNA testing, with results available within 45 min, was later recognised as a critical diagnostic tool in the pathology-led, national COVID-19 testing framework for the detection of the first case in remote First Nations communities [1].

The program has been briefly conceptualised as follows. By the end of August 2020, 72,624 SARS-CoV-2 RNA tests had been performed with most (67.1%) testing remote First Nations peoples and resulting in approximately 6% positivity [4]. By September 2020, the COVID-19 POC Testing Program had scaled up to 86 remote health clinics servicing predominantly First Nations peoples. By 31 August 2022, at capacity, the COVID-19 POC Testing Program had scaled up across six Australian jurisdictions to comprise 105 enrolled health clinics (78% located in remote or very remote communities) with a median aerial distance of 569 km, or eight hours drive, from the nearest COVID-19 testing laboratory [1]. POC device operation was facilitated by more than 900 local, trained and competent primary care staff [1].

An external review of the COVID-19 POC Testing Program considered it highly successful. It was reported that the program supported timely community-led public health responses and contributed to the estimation of between 23,000 and 122,000 COVID-19 infections averted within 40 days of the first infection in a remote community [4]. This equated to health system cost savings estimated between AU$337 million and AU$1.8 billion in the first 40 days, due to avoided hospitalisation and associated negative impacts on quality of life [4]. Importantly, the review also highlighted key foundational processes that enabled SARS-CoV-2 RNA testing within the COVID-19 POC Testing Program to be conducted safely and to standards comparable to those achieved in laboratory testing environments. These factors included the rigorous training of testing operators, managed analytical quality, thorough and regular internal quality control (QC) and external quality assurance and a low (0.7%) rate of unsuccessful tests [4].

The COVID-19 POC Testing Program aligned External Quality Assessment (EQA) processes with national and international requirements. The National Pathology Accreditation Advisory Council (NPAAC) requirements for POC testing in accredited pathology laboratories mandate enrolment in an available EQA program to assess the reliability and accuracy of test results. In this context, participation in an EQA program ensures the performance of testing using the POC device is acceptable when compared to the POC testing service’s risk analysis [5]. A similar rationale for the utilisation of EQA programs in laboratory testing has been noted by the World Health Organization (WHO), with EQA program results allowing comparisons between testing sites, the identification of training needs and potential analytical problems or processes that require improvement [6].

This paper describes the implementation of the customised SARS-CoV-2 EQA program at health services enrolled in the COVID-19 POC Testing Program, and reports for the first time on the analytical performance observed in this setting during the first two years of operation, from July 2020 to July 2022. More broadly, these findings aim to illustrate the global importance of high-quality, readily available and non-cost-prohibitive EQA programs for improving molecular-based POC testing for infectious diseases.

## 2. Methods

### 2.1. Molecular SARS-CoV-2 POC Testing Network

From April 2020, molecular-based SARS-CoV-2 POC testing using GeneXpert^®^ (Cepheid, Sunnyvale, CA, USA) was implemented in phases at primary healthcare services across six Australian jurisdictions. By September 2020, up to 86 remote health clinics were enrolled in the COVID-19 POC Testing Program with program capacity, 105 enrolled health clinics, reached by August 2022. Details of the Aboriginal and Torres Strait Islander COVID-19 POC testing network have been reported elsewhere [1,7].

### 2.2. SARS-CoV-2 POC Testing Operator Training and Competency

Non-scientific, clinical staff from enrolled health services completed publicly available online education in hand hygiene, personal protective equipment (PPE) donning and doffing, infection control and work health and safety to be eligible for SARS-CoV-2 RNA POC testing operator training. Details of the SARS-CoV-2 RNA POC testing training resources developed by the program team, including specimen collection/test process risk assessments, standard operating procedures, visual training/educational posters, training presentations and verification of the inactivating molecular transport media [8] have been previously reported. Due to the pandemic-imposed interstate and local travel restrictions, SARS-CoV-2 RNA POC testing operator training was delivered by program staff members using Teams/Webex online platforms. Briefly, the SARS-CoV-2 RNA POC testing operator training comprised (a) theoretical training and assessment, (b) practical training and competency assessment using SARS-CoV-2 positive and negative QC testing and (c) EQA training [1]. Operators were issued unique POC device logins and passwords and were deemed competent for a period of 12 months. Ongoing operator support was provided via program-specific technical assistance and result interpretation support phone services. Operators were re-trained following significant changes to program standard operating procedures, identified quality corrective actions or competency expiry.

### 2.3. EQA Survey Organisation and Samples

The Royal College of Pathologists of Australasia Quality Assurance Program (RCPAQAP, St Leonards, NSW, Australia) manufactured the SARS-CoV-2 RNA EQA material. Simple optimised EQA product specifications and processes were designed collaboratively by the RCPAQAP and COVID-19 POC Testing Program scientific teams for ease of use by non-scientific, health service staff (Table 1). Briefly, each EQA survey consisted of two samples for testing: a SARS-CoV-2 positive sample (of variable, inter-survey viral loads) inactivated by gamma irradiation and a negative sample (containing Madin-Darby canine kidney (MDCK) cells). The material was suspended in 800 µL of buffered saline solution to simulate a respiratory sample. Homogeneity and stability testing of the EQA material was conducted by the RCPAQAP using an in-house process. An external laboratory validated the content of each sample before dispatch (Table 2).

The first EQA testing survey was issued in July 2020 across six Australian jurisdictions to the 80 eligible primary care health services in the COVID-19 POC Testing Program. Six subsequent EQA surveys were issued approximately every 3–4 months throughout 2020, 2021 and 2022 to eligible primary care health services (with exact numbers shown in Table 3). Some enrolled sites were ineligible to participate in specific EQA surveys due to delayed operator training/competency post-device installation, staff shortages during periods of redeployment to COVID-19 outbreak areas, staff illness, technical device maintenance/troubleshooting and/or instances of device repair.

### 2.4. EQA Dispatch and Storage

Updated lists of eligible health service details were provided to the RCPAQAP prior to each survey dispatch. Specimens were shipped at ambient temperature. Operators were instructed to test the EQA specimens immediately on arrival or freeze the material at −20 °C until testing.

### 2.5. Testing of EQA Specimens and Result Interpretation

The operators were blinded to EQA results at the time of testing, consistent with usual EQA procedures. Samples were tested on the GeneXpert^®^ using the Cepheid Xpert Xpress SARS-CoV-2 assay. The assay detected two SARS-CoV-2 gene targets: the nucleocapsid (N2 gene) and the envelope (E gene) and included an in-built Sample Processing Control (SPC). For detected gene targets, cycle threshold (Ct) values were recorded within the device software, but consistent with the on-label instructions for assay use, operators submitted qualitative (i.e., positive, presumptive positive or negative) SARS-CoV-2 RNA EQA results only. SARS-CoV-2 RNA was ‘positive’ if the N2 gene was detected in either the presence or absence of the E gene. A ‘presumptive positive’ SARS-CoV-2 result was reported if the E gene was detected without the N2 gene. A ‘negative’ result was reported if neither the N2 nor E genes were detected.

EQA testing was reported as ‘not assessed’ if ‘no result/s was submitted’ by the site or the test was ‘unsuccessful’. Unsuccessful tests were sub-categorised by the GeneXpert Dx software (Version 5.2 (initial sites), with upgrade to Versions 6.2 and later 6.3 across the included EQA cycles) as ‘invalid’, ‘error’ or ‘no result’. An ‘invalid’ was reported if the in-built Sample Processing Control (SPC) failed, the sample was not properly processed, PCR was inhibited or the sample was inadequate. An ‘error’ was indicative of an internal Probe Check Control (PCC) fail and subsequent assay abortion due to either an improperly filled reaction tube, a detected reagent probe integrity problem, exceeded pressure limits or valve positioning error. A ‘no result’ indicated that collected device data were insufficient, such as in circumstances where the operator stopped a test in progress.

### 2.6. Submission and Assessment of Results

Initially, the results were manually transcribed from the device to paper-based program-specific result forms by the operator and submitted to the COVID-19 POC Testing Program Help Desk for collation prior to analysis and reporting by the RCPAQAP. The results submission process was later adapted to online entry by the operator via the COVID-19 POC Testing Program website. Submitted results were assessed as ‘concordant’ if they were consistent with the expected qualitative result and ‘discordant’ if they were inconsistent with the expected qualitative result.

### 2.7. EQA Participant Reports

After each EQA survey, customised generic and individual site reports were issued by the RCPAQAP. Individual sites received reports summarising site-specific analytical performance compared to other deidentified services enrolled in the COVID-19 POC Testing Program. EQA reports provided to enrolled health services were appropriate for interpretation for a layperson, whereas the generic reports provided to the COVID-19 POC Testing Program team included standardised EQA graphs and analyses and inter-site gene target cycle threshold (Ct) monitoring.

### 2.8. Corrective Action and Re-Training

Discordant results were investigated by the COVID-19 POC Testing Program Help Desk and corrective actions were documented, but initial result submissions were not modified. The COVID-19 POC Testing Program Help Desk facilitated the dispatch of additional EQA specimens from the RCPAQAP for corrective actions as required. Root cause analysis and corrective actions were recorded and reported to the COVID-19 POC Testing Program Quality Committee (once established) and Clinical Advisory Group.

## 3. Results

### 3.1. Participating Health Services

Seven SARS-CoV-2 RNA POC testing EQA surveys were completed from mid-2020 to mid-2022, with the number of eligible sites ranging from 80 (Survey 1) to 89 (Survey 6). Eligible site EQA participation was greater than 90% for all surveys, ranging from 93.0% (Survey 2) to 100% (Survey 3) (Table 3).

### 3.2. Quality Assurance Performance

#### 3.2.1. Overall EQA Performance

**Figure 1:**
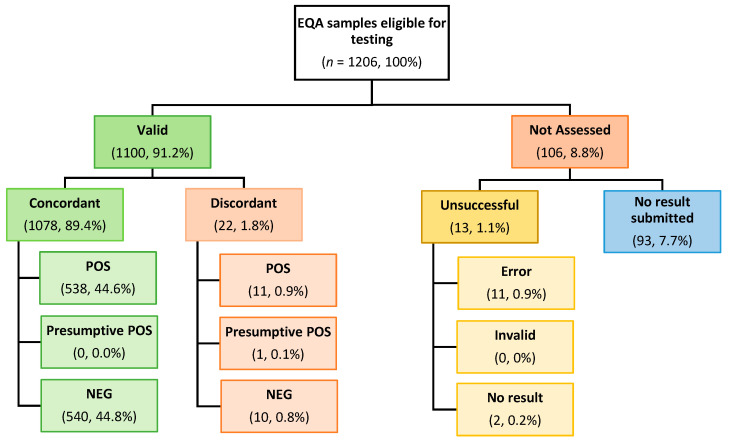
Flowchart of inter-site and inter-survey (Surveys 1 to 7) EQA testing for SARS-CoV-2. Values in each box are shown as number, percentage (%) of total inter-site and inter-survey specimens tested.

A total of 1206 EQA samples were issued to eligible sites for Surveys 1 to 7, with 91.2% (*n* = 1100) valid results reported. Concordance across all eligible survey samples was 89.4% (*n* = 1078), with the frequency of positive (*n* = 538) and negative (*n* = 540) EQA results submitted consistent with that expected from the pre-issue testing (Figure 1). Adjusting overall EQA concordance (*n* = 1078) for the total number of samples with submitted valid results (*n* = 1100), the analytical performance was high, with concordance at 98%. Over Surveys 1 to 7, 8.8% (*n* = 106) of results were not assessed, with the majority attributed to no result being submitted by the health service (Figure 1). The overall unsuccessful test (i.e., ‘invalid’, ‘error’ and ‘no result’) rate was low (1.1%, *n* = 13), with most unsuccessful tests attributed to device error (Figure 1).

#### 3.2.2. Inter-Site Performance by Specimen

The median inter-site result concordance was 90.2%, with the highest inter-site overall concordance reported (97.5%) in Survey 2 for specimen MCoV-2020-04 and the lowest inter-site overall concordance (80.0%) in Survey 1 for specimen MCoV-2020-01 (Table 4). The median inter-site result discordance across all specimens was 1.2%, with the highest inter-site discordance reported in Survey 1 for specimen MCoV-2020-01 (7.5%, *n* = 6) and specimen MCoV-2020-02 (6.3%, *n* = 5), but it was then less than 2.3% across all other surveys and specimens (Table 4). Over Surveys 1 to 7, the percentage of inter-site specimens that were not assessed ranged from 5.7% in Survey 4 for specimens MCoV-2021-03 and MCoV-2021-04 to 12.5% in Survey 1 for specimen MCoV-2020-01 (Table 4).

#### 3.2.3. Reporting of Discordant Results

Of the total 1206 EQA specimens tested, there were 11 false positive results (0.9%), 1 presumptive positive result (0.08%) and 10 false negative results (0.8%) reported. The rate of false positive result discordance was highest in Survey 1 (6.2% for specimen MCoV-2020-02). Lower rates of false positive result discordance were reported in Survey 2, specimen MCoV-2020-03 (1.3%); Survey 3, specimen MCoV-2021-02 (1.1%); Survey 4, specimen MCoV-2021-03 (2.3%); Survey 5, specimen MCoV-2021-06 (1.1%); and Survey 7, specimen MCoV-2022-03 (1.2%) (Table 5). Similarly, false negative EQA results with the highest discordance were reported for Survey 1, specimen MCoV-2020-01 (7.5%), but occurred at lower rates in Survey 2, specimen MCoV-2020-04 (1.2%); Survey 4, specimen MCoV-2021-04 (2.3%); and Survey 5, specimen MCoV-2021-05 (1.1%) (Table 5). The only presumptive positive result was a false positive for Survey 6, specimen MCoV-2022-02 (Table 5).

#### 3.2.4. Post-Report Corrective Actioning of Discordant Results

**Figure 2:**
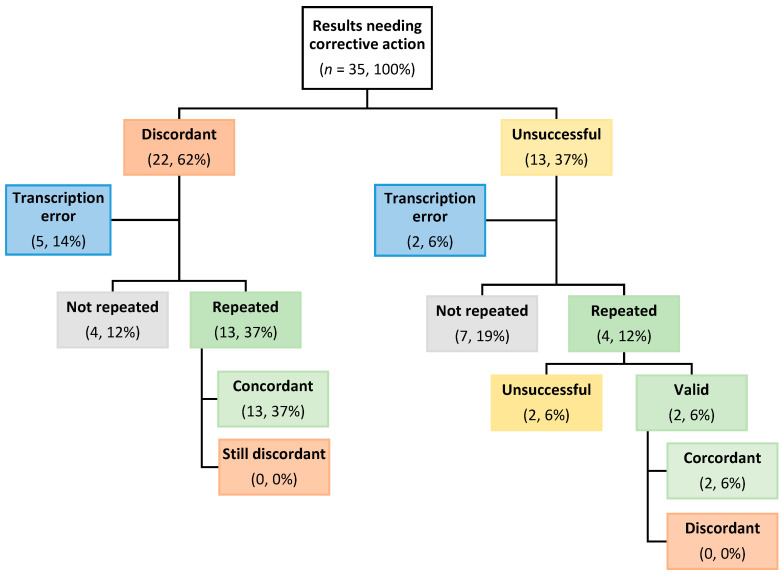
Flowchart of post-report EQA results (Surveys 1 to 7) requiring corrective action. Values in each box are shown as number, percentage (%) of total inter-site and inter-survey EQA specimens tested.

Of the 35 results requiring post-report corrective action, 22 were discordant and 13 were unsuccessful. Manual transcription error was associated with 5 of the discordant results and 2 of the unsuccessful results (Figure 2) and led to operator re-training. For root cause analyses, corrective action and quality management documentation purposes only, 13 of 17 specimens initially reported as discordant, excluding those attributed to transcription errors, were re-tested by each of the operators, with all results concordant on repeat (Figure 2). Of the 13 specimens tested unsuccessfully, 4 were re-tested by each of the operators, and 2 gave valid concordant results on repeat. Only 2 of the repeated EQA specimens were unsuccessful on the second attempt; however, 7 specimens were not re-tested (Figure 2).

## 4. Discussion

Early in the COVID-19 pandemic, rapidly developed SARS-CoV-2 molecular-based in vitro diagnostics were new to the market and approved for use by the Therapeutic Goods Administration (TGA) in Australia under emergency use only (EUO) conditions. Under these circumstances, both internal QC materials and EQA programs for SARS-CoV-2 RNA were critical for post-market surveillance of rapidly introduced COVID-19 assays, as well as the ongoing assessment of SARS-CoV-2 RNA assay quality in accredited laboratories [9,10,11,12] and at the point-of-care. In the COVID-19 POC Testing Program, collaboration with an accredited EQA provider, the RCPAQAP, facilitated the prompt design and manufacture of fully customised, homogenous and stable SARS-CoV-2 RNA EQA materials, instructions for use, result submission and reporting processes. This enabled an easy-to-use EQA program suitable for non-scientific, POC testing operators to be rapidly implemented. This study is the first to report SARS-CoV-2 RNA EQA POC test results from a primary care health service network in Australia.

The first SARS-CoV-2 RNA EQA survey was issued for this network in July 2020. Participation in the SARS-CoV-2 RNA EQA POC testing program was high across all seven surveys, although the number of eligible remote health services enrolled in the COVID-19 POC Testing Program increased as molecular-based POC testing was scaled up across Australia. Diligent remote POC testing operators, comprehensive operator training and competency assessment processes and the ongoing operator support provided via the COVID-19 POC Testing Program Help Desk and result support Hotline likely contributed to the observed active participation in EQA. Factors including delayed operator training/competency post-device installation, staff shortages during periods of redeployment to COVID-19 outbreak areas, staff illness, technical device maintenance/troubleshooting and instances of device repair resulted in some enrolled sites being ineligible to participate in specific EQA surveys. Similarly, limited workforce capacity for POC testing at remote primary care health care services is likely to have contributed to several sites not returning EQA results, which was observed at a consistent rate across the seven surveys and warrants further investigation for improvement.

The high overall test validity and low error rate were indicative of the competency of health service staff to perform SARS-CoV-2 RNA POC testing on the GeneXpert, despite the staggered program site scale-up requiring high ongoing training needs and resulting in newly trained GeneXpert operators participating in the EQA program for the first time during different EQA surveys. Overall, a small number of EQA tests were unsuccessful (i.e., ‘invalid’, ‘error’ and ‘no result’), with the majority attributed to GeneXpert internal Probe Check Control (PCC) failure and subsequent assay abortion. The device manufacturer identified this error occurring from an improperly filled test cartridge reaction tube, detected reagent probe integrity problem, exceeded pressure limits or valve positioning error, but the frequency of this error did not warrant concern in this program.

Similar to the overall performance in a SARS-CoV-2 RNA EQA survey reported for 12 Australian and 4 New Zealand laboratories in March 2020 [9], the overall SARS-CoV-2 RNA POC testing performance at eligible remote health services improved following the first EQA survey. In this study, the inclusion of both negative and positive QC and EQA materials, combined with the similar concordance between expected and reported results of qualitatively positive and negative SARS-CoV-2 EQA specimens, provided rapid insight into two operational root causes of error. The first related to a GeneXpert result software default, which displayed the last SARS-CoV-2 RNA result viewed by the operator on the screen, rather than overriding this with the SARS-CoV-2 RNA result derived from the last specimen tested. The second was associated with the manual transcription error of the EQA result on the paper (or later, online) result submission sheet. Although it is noted that SARS-CoV-2 RNA patient results in the COVID-19 POC testing program were electronically transferred from the device to the health service patient management system and the state/territory public health teams, intensive education and ongoing support for remote POC testing operators to confirm correct specimen identification prior to verbally communicating results was provided, especially in the early stages of the program. Whilst in this study, the EQA results initially submitted to the RCPAQAP were not amended, it is important to note that all EQA specimens that underwent corrective action re-testing by POC operators were reported as concordant. Collectively, these observations highlight the value of quality management practices in the identification of errors in real-world testing environments and the benefits of ongoing participation in EQA programs.

Although the COVID-19 viral load varied and different variants of concern were included in EQA material (as they became available), interestingly, only a single EQA test performed in Survey 6 for specimen MCoV-2022-02 was reported as a presumptive positive result. For this specimen, the E gene target was detected with a high cycle threshold (Ct) of 37.4, despite the specimen having an expected negative result. The limit of detection of the GeneXpert SARS-CoV-2 assay was low (0.0200 PFU/mL); however, it is noted that the process for managing presumptive positive patient results included repeat testing until a qualitative positive or negative result was obtained or the specimen was sent to the laboratory for testing on an alternative platform. Furthermore, subsequent development of the 4-plex GeneXpert respiratory multiplex assay, which tests for SARS-CoV-2, Influenza A, Influenza B and Respiratory Syncytial Virus, eliminated the presumptive positive call out as the number of reading channels for the fluorescent signals does not allow for separated N2 and E gene target Ct reporting. Future SARS-CoV-2 EQA programs may therefore benefit from further challenging POC operator competency by including viral loads closer to the assay limit of detection and randomly allocating positive or negative specimens to the survey cycles.

## 5. Conclusions

This is the first report of SARS-CoV-2 POC testing performance in primary care settings in Australia, assessed using standard proficiency testing programs for EQA. These findings demonstrate the capability of trained and competent non-scientific staff to successfully perform nucleic acid amplification testing for SARS-CoV-2 in primary care settings. More broadly, these findings highlight an ongoing need for well-designed, cost-effective, accredited EQA programs for other molecular-based infectious disease POC testing programs globally, which can be used to assess the reliability and accuracy of test results and ensure the performance of testing using the device is acceptable.

## Figures and Tables

**Table 1:** Optimised EQA specifications and processes.

EQA Specification	Description
Deactivated material	Gamma irradiated to allow safe product transportation and reduce infectivity risk to operator
Liquid-stable	No reconstitution required by operator. Vial size facilitated safe handling with the assay manufacturer’s sampling pipette (i.e., no additional equipment required)
Temperature-stable	Product shipped at ambient temperature
Variable viral loads	Survey included negative and positive specimens, with positive viral loads varied across survey production
Inclusion of variants of concern	Delta and Omicron variants of concern were included in EQA specimen manufacture as they became prominent through the pandemic
Sample volume	Sample volume was adequate to allow for repeat testing, if required for corrective action
Vial identification	Survey-specific coloured vial lids for easy specimen number identification
Instructions for Use	Printed, customised layperson instructions for use, with reference to colour coded specimens
Survey Frequency	Minimised to two samples per survey at quarterly intervals to reduce burden on remote health service staff
Dispatch Lists	Customised survey-specific product dispatch lists to maximise successful delivery to remote health services
Result Submission	Options for simple, program-specific, paper-based (and later) web-based result submission using the POC testing resources or website with the same operator ID and password
Reports	Simplified, layperson feedback reports for health services

**Table 2:** Summary of pre-issue results for EQA Surveys 1–7.

Survey Number and Issue Date (Month, Year)	Specimen ID	Specimen Constituent	SARS-CoV-2 Gene Target/s and Expected Cycle Threshold (Ct)	Expected Specimen Qualitative Result
1 (Jul 2020)	MCoV-2020-01	Coronavirus (SARS-CoV-2)	E gene Ct 17.7 N2 gene Ct 20.2	Positive
	MCoV-2020-02	MDCK—Negative	E gene Ct 0 N2 gene Ct 0	Negative
2 (Nov 2020)	MCoV-2020-03	MDCK—Negative	E gene Ct 0 N2 gene Ct 0	Negative
	MCoV-2020-04	Coronavirus (SARS-CoV-2)	E gene Ct 21.3 N2 gene Ct 23.8	Positive
3 (Feb 2021)	MCoV-2021-01	Coronavirus (SARS-CoV-2)	E gene Ct 21.7 N2 gene Ct 24.4	Positive
	MCoV-2021-02	MDCK—Negative	E gene Ct 0 N2 gene Ct 0	Negative
4 (Jun 2021)	MCoV-2021-03	MDCK—Negative	E gene Ct 0 N2 gene Ct 0	Negative
	MCoV-2021-04	Coronavirus (SARS-CoV-2)	E gene Ct 25.8 N2 gene Ct 28.3	Positive
5 (Sep 2021)	MCoV-2021-05	Coronavirus (SARS-CoV-2)	E gene Ct 26.3 N2 gene Ct 28.7	Positive
	MCoV-2021-06	MDCK—Negative	E gene Ct 0 N2 gene Ct 0	Negative
6 (Feb 2022)	MCoV-2022-01	Coronavirus (SARS-CoV-2 Delta variant)	E gene Ct 27.6 N2 gene Ct 30.2	Positive
	MCoV-2022-02	MDCK—Negative	E gene Ct 0 N2 gene Ct 0	Negative
7 (May 2022)	MCoV-2022-03	MDCK—Negative	E gene Ct 0 N2 gene Ct 0	Negative
	MCoV-2022-04	Coronavirus (SARS-CoV-2 Omicron variant)	E gene Ct 29.3 N2 gene Ct 31.6	Positive

**Table 3:** Participation rates (%) of eligible sites across EQA Surveys 1 to 7.

Survey	1		2		3		4		5		6		7	
Sample	2020-01	2020-02	2020-03	2020-04	2021-01	2021-02	2021-03	2021-04	2021-05	2021-06	2022-01	2022-02	2022-03	2022-04
Number of eligible sites	80	80	86	86	88	88	88	88	87	87	89	89	85	85
% of eligible sites submitted results	96%	95%	93%	93%	100%	100%	97%	97%	99%	99%	94%	94%	94%	94%

**Table 4:** The percentage (%) of inter-site concordance (green), discordance (red) and not-assessed (orange) rates from eligible sites for Surveys 1 to 7.

Survey	Specimen ID	Expected Result	Inter-Site Concordance % (*n*)	Inter-Site Discordance % (*n*)	Inter-Site Not Assessed % (*n*)
1 (Jul 2020)	MCoV-2020-01	Positive	80.0% (64)	7.5% (6)	12.5% (10)
	MCoV-2020-02	Negative	83.7% (67)	6.3% (5)	10.0% (8)
2 (Nov 2020)	MCoV-2020-03	Negative	89.5% (77)	1.2% (1)	9.3% (8)
	MCoV-2020-04	Positive	90.7% (78)	1.2% (1)	8.1% (7)
3 (Feb 2021)	MCoV-2021-01	Positive	90.9% (80)	0.0% (0)	9.1% (8)
	MCoV-2021-02	Negative	89.8% (79)	1.1% (1)	9.1% (8)
4 (Jun 2021)	MCoV-2021-03	Negative	92.0% (81)	2.3% (2)	5.7% (5)
	MCoV-2021-04	Positive	92.0% (81)	2.3% (2)	5.7% (5)
5 (Sep 2021)	MCoV-2021-05	Positive	89.7% (78)	1.1% (1)	9.2% (8)
	MCoV-2021-06	Negative	90.8% (79)	1.2% (1)	8.0% (7)
6 (Feb 2022)	MCoV-2022-01	Positive	91.0% (81)	0.0% (0)	9.0% (8)
	MCoV-2022-02	Negative	89.9% (80)	1.1% (1)	9.0% (8)
7 (May 2022)	MCoV-2022-03	Negative	90.6% (77)	1.2% (1)	8.2% (7)
	MCoV-2022-04	Positive	89.4% (76)	0.0% (0)	10.6% (9)
Median (Interquartile (IQR) range)			90.2% (89.6–90.9%)	1.2% (1.1–2.0%)	9.0% (8.2–9.3%)

**Table 5:** Inter-site performance for Surveys 1 to 7. Reported as positive (POS), Presumptive Positive (PP) or Negative (NEG) results. SARS-CoV-2 results concordance (green), discordance (orange), unsuccessful (yellow) or no result submitted (blue).

Survey Number	Specimen ID	Expected Result	POS % (*n*)	PP % (*n*)	NEG % (*n*)	Unsuccessful % (*n*)	No Result Submitted% (*n*)
1 (Jul 2020)	MCoV-2020-01	Positive	80.0% (64)	0.0% (0)	7.5% (6)	3.8% (3) Error, 1.2% (1) No Result	7.5% (6)
	MCoV-2020-02	Negative	6.2% (5)	0.0% (0)	83.8% (67)	1.2% (1) Error	8.8% (7)
2 (Nov 2020)	MCoV-2020-03	Negative	1.2% (1)	0.0% (0)	89.5% (77)	1.2% (1) Error	8.1% (7)
	MCoV-2020-04	Positive	90.7% (78)	0.0% (0)	1.2% (1)	0.0% (0)	8.1% (7)
3 (Feb 2021)	MCoV-2021-01	Positive	90.9% (80)	0.0% (0)	0.0% (0)	1.1% (1) Error	8.0% (7)
	MCoV-2021-02	Negative	1.1% (1)	0.0% (0)	89.8% (79)	1.1% (1) No Result	8.0% (7)
4 (Jun 2021)	MCoV-2021-03	Negative	2.3% (2)	0.0% (0)	92.0% (81)	0.0% (0)	5.7% (5)
	MCoV-2021-04	Positive	92.0% (81)	0.0% (0)	2.3% (2)	0.0% (0)	5.7% (5)
5 (Sep 2021)	MCoV-2021-05	Positive	89.7% (78)	0.0% (0)	1.1% (1)	1.1% (1) Error	8.1% (7)
	MCoV-2021-06	Negative	1.1% (1)	0.0% (0)	90.8% (79)	0.0% (0)	8.1% (7)
6 (Feb 2022)	MCoV-2022-01	Positive	91.0% (81)	0.0% (0)	0.0% (0)	0.0% (0)	9.0% (8)
	MCoV-2022-02	Negative	0.0% (0)	1.1% (1)	89.9% (80)	0.0% (0)	9.0% (8)
7 (May 2022)	MCoV-2022-03	Negative	1.2% (1)	0.0% (0)	90.5% (77)	1.2% (1) Error	7.1% (6)
	MCoV-2022-04	Positive	89.4% (76)	0.0% (0)	0.0% (0)	3.5% (3) Error	7.1% (6)

## Data Availability

The data will be made available upon reasonable request.
